# Natural history of sleep before and after total joint arthroplasty is poor and unimproved by melatonin: A randomized controlled study

**DOI:** 10.1051/sicotj/2026029

**Published:** 2026-09-15

**Authors:** Alex Lancaster, Brenna E. Blackburn, Shanna Loughmiller, Michael J. Archibeck, Jeremy M. Gililland, Lucas A. Anderson

**Affiliations:** 1 Department of Orthopaedic Surgery, University of Utah 590 Wakara Way Salt Lake City UT 84108 USA; 2 Orthopedic Surgerym, St. Charles Health System 1342 NE Medical Center Drive Bend OR 97701 USA

**Keywords:** Sleep, Total knee arthroplasty, Total hip arthroplasty, Melatonin

## Abstract

*Introduction*: Poor sleep has been shown to affect surgical recovery with negative impacts on function and pain. Osteoarthritis can negatively impact sleep, as can surgery, making arthroplasty patients especially at risk for these ill effects both before and after surgery. Our study goals were to monitor sleep recovery and determine if perioperative melatonin could improve sleep quality compared to a placebo. *Methods*: From 2020 to 2023, a cohort of 48 primary total hip arthroplasty (THA) and total knee arthroplasty (TKA) patients were randomized to melatonin (*n* = 23) or placebo (*n* = 25) for 7 weeks (1 week preoperatively, 6 weeks postoperatively). Patients were excluded if they already used sleep aids or had a sleep disorder. Sleep questionnaires collecting daily sleep and opioid data were electronically administered pre- and postoperatively up to 3 months. Generalized estimating equations were used to assess longitudinal sleep and opioid use. *Results*: THA patients in the melatonin group had significantly more hours of sleep than the controls, with the numbers available for this study (*p* = 0.009). However, this did not translate to improved sleep scores overall. There were no significant differences between the melatonin and control groups in the TKA patients. Sleep quality scores significantly improved over time for both hip and knee patients (*p* = 0.03). For THA patients, sleep scores did not return to baseline until 3 months postoperatively, whereas scores for TKA patients had improved beyond baseline by 2 weeks postoperatively. *Conclusion*: Sleep disturbance is ubiquitous prior to surgery, and quality and quantity are negatively impacted after TJA surgery as well. TKA patients’ sleep improved by 2 weeks, whereas THA patients did not improve till 3 months after surgery. The addition of perioperative melatonin led to more hours of sleep for THA patients but did not otherwise positively impact sleep quality, opioid use, or recovery in primary TJA patients.

## Introduction

The volume of total joint arthroplasty (TJA) surgery in the United States continues to increase, and clinicians continue to search for ways to improve overall outcomes by addressing issues in the perioperative period [1–3]. In recent years, sleep has become an increasingly popular target for interventions. Sleep is known to be critical to proper functioning and has many restorative benefits. Poor sleep has been shown to negatively affect many key aspects related to surgical recovery, including immune function, glucose control, cognitive function, pain, length of stay, physical function, opioid consumption, and mental health [4–10].

Many osteoarthritis patients have sleep disturbances prior to surgery [11]. Sleep quality has been shown to be further reduced following orthopedic surgery [11–13]. The importance of sleep and the known disturbances for arthroplasty patients prompted further research into possible interventions. Melatonin, a naturally occurring substance that regulates sleep/wake cycles, is a popular sleep aid due to its excellent safety profile and low cost [14]. Several studies from other surgical fields have examined the use of melatonin in postoperative patients and have shown some promising improvements in sleep quality and a decrease in opioid use [7, 15–17]. At the outset of our study, there were few publications in orthopedics specifically investigating perioperative melatonin use [18].

The purpose of this study was to determine if the addition of longer duration perioperative melatonin, along with sleep hygiene training for elective TJA patients, could improve sleep scores, improve patient outcomes, and/or decrease opioid use compared to placebo. Longer-term general sleep data were also collected to better characterize the perioperative natural history of sleep for TJA patients. Our hypothesis was that melatonin would be beneficial in improving sleep scores and that sleep dysfunction would persist in postoperative patients for many weeks.

## Material and methods

This single-center, double-blinded, randomized controlled trial enrolled patients undergoing unilateral total hip or knee arthroplasty. The primary aim of the study was to determine if there was a significant difference in hours and quality of sleep between the melatonin and control cohorts in TJA patients. The secondary aim of the study was to characterize the natural history of sleep disturbance after TJA. Patients were excluded if they were already using a sleep aid or melatonin, if they had a previously diagnosed sleep disorder, alcohol or drug abuse, or use of calcium channel blockers. Patients undergoing staged bilateral surgery, with the second surgery being done within 3 months of the first surgery, were also excluded. This study was approved by the Institutional Review Board.

Once enrolled, patients were randomized to either the placebo or melatonin arm (3 mg of melatonin) using a simple randomization scheme via a computer random number generator by the biostatistician on the study. Study medications were obtained from the University of Utah Investigational Research Pharmacy and were in bottles with no identification to patients of which medication they received. Only the study coordinators who enrolled patients were aware of which medication each patient was randomized to; both the patients and the healthcare team were blinded.

All patients received the same instructions regardless of randomization group. Patients were instructed to take one pill before bedtime, starting 1 week before surgery and continuing through 6 weeks after surgery on an outpatient basis. Patients were asked to complete surveys throughout the study period, which included daily surveys asking about narcotic use, hours of sleep, and quality of sleep (1–10 scale) from the previous night. These included the Pittsburgh Sleep Quality Index (PSQI), the Stanford Sleepiness Scale (SSS), a question on sleep quality, and VAS pain scores, which were given preoperatively, at 2 weeks, 6 weeks, and 3 months postoperatively. The PSQI was the primary outcome and is the most commonly used, validated sleep questionnaire, with a lower score reflecting higher quality sleep [19]. The SSS is a scale on which patients fill out their level of sleepiness on a Likert scale from 1–7 for each hour of the day they are awake, with 1 being wide awake and 7 being sleep onset soon. The scores were summed to give an overall score of sleepiness throughout the day, with a lower score being less sleepy when awake. The sleep quality question was a 0–10 scale (0 being terrible and 10 being excellent) on sleep quality for the previous 7 nights. All study patients, regardless of study arm, were given a handout on sleep hygiene.

An a priori power analysis showed that we needed 64 patients per group to identify a difference of 30 min of sleep between the two groups. To account for patient attrition, our goal was 75 patients per group. Demographics were compared between the melatonin and placebo groups using *chi*-square and t-tests. Distributions of sleep metrics were checked for normality. *T*-tests were used to compare sleep metrics between the melatonin and placebo groups at each time point. Generalized estimating equations were utilized to model the longitudinal data from the daily questionnaires (hours of sleep per night, quality of sleep per night, and number of narcotic pills taken per day) between the melatonin and placebo groups. Models were adjusted for age, body mass index (BMI), and baseline pain. Statistical analysis was completed using SAS 9.4 (Cary, NC) and Stata 17 (College Station, TX).

## Results

**Figure 1:**
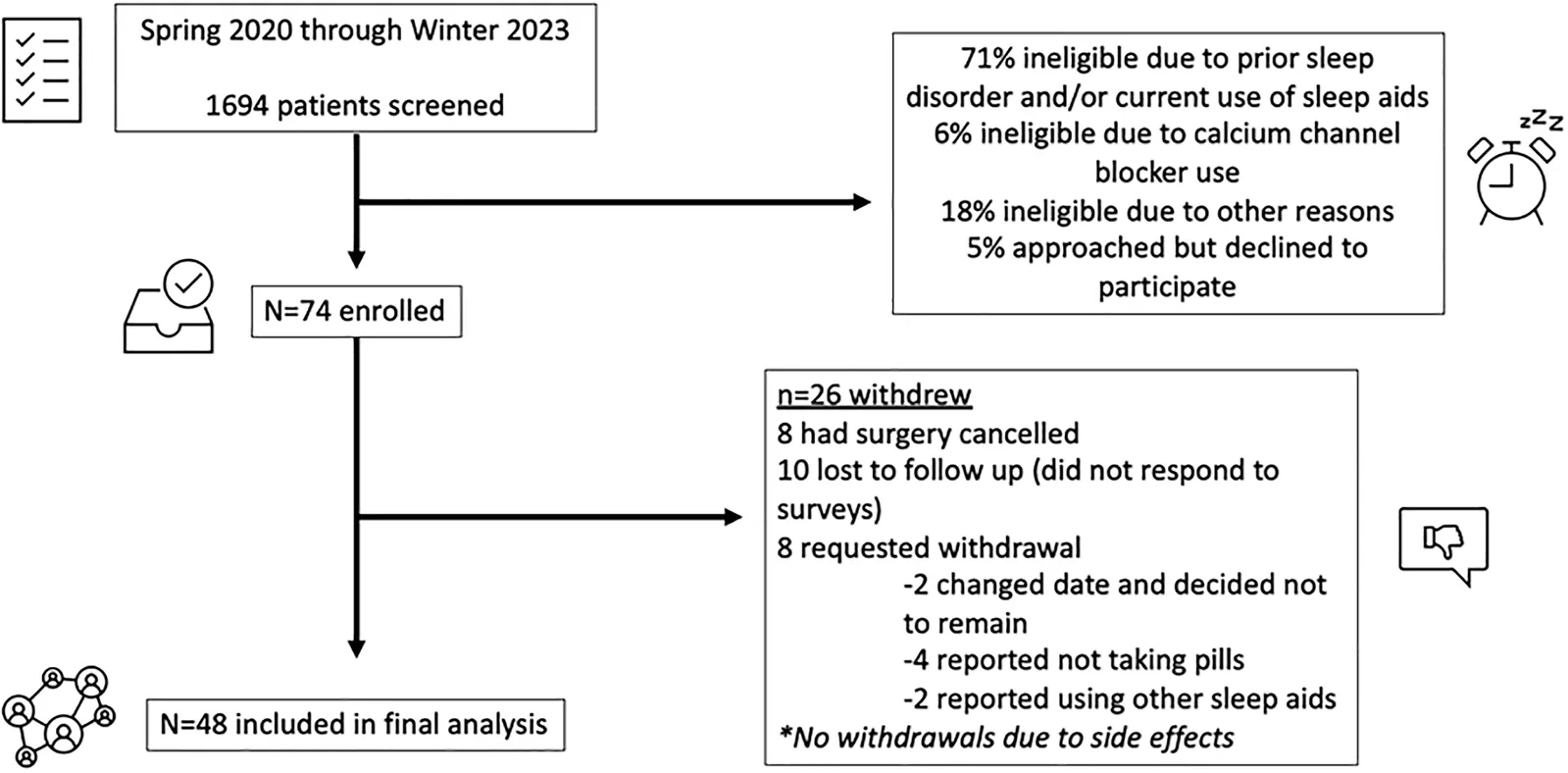
CONSORT diagram.

Due to the higher-than-expected rate of patients excluded, we were not able to meet the study’s enrollment goal. While over 1600 patients were screened, more than 90% of patients who underwent a THA or TKA were ineligible to be enrolled, most commonly due to pre-existing sleep disorders or current use of sleep aids (Figure 1). Overall, 74 patients were enrolled in the study, with 26 patients being withdrawn. A total of 48 patients were included in the final analysis, 23 in the melatonin group and 25 in the placebo group. There were no significant differences at baseline between the two groups with regard to age, BMI, sex, joint, or pain. Patients in the melatonin group did have significantly lower PSQI score at baseline to start (5.6 vs 8.0, *P* = 0.0137) (Table 1). Both groups had average preoperative PSQI scores greater than 5, indicating baseline “poor” sleep.

**Table 1:** Demographics.

	Melatonin (*N* = 23)	Placebo (*N* = 25)	
	Mean (std)	Mean (std)	*p*-value
Age	63.3 (9.3)	60.6 (11.4)	0.3801
Body mass index	28.7 (6.4)	29.8 (6.4)	0.5657
Pain prior to surgery (0–10)	4.6 (2.1)	4.8 (2.6)	0.8022
Sleep quality prior to surgery (0–10)	7.0 (1.8)	6.1 (2.3)	0.1767
Preoperative PSQI	5.6 (2.6)	8.0 (3.3)	0.0137
	*N* (%)	*N* (%)	*p*-value
Sex			
Female	13 (56.5)	18 (72.0)	0.2627
Male	10 (43.5)	7 (28.0)	
Joint			
Hip	10 (43.5)	14 (56.0)	0.3861
Knee	13 (56.5)	11 (44.0)	

PSQI: Pittsburgh Sleep Quality Index.

**Figure 2:**
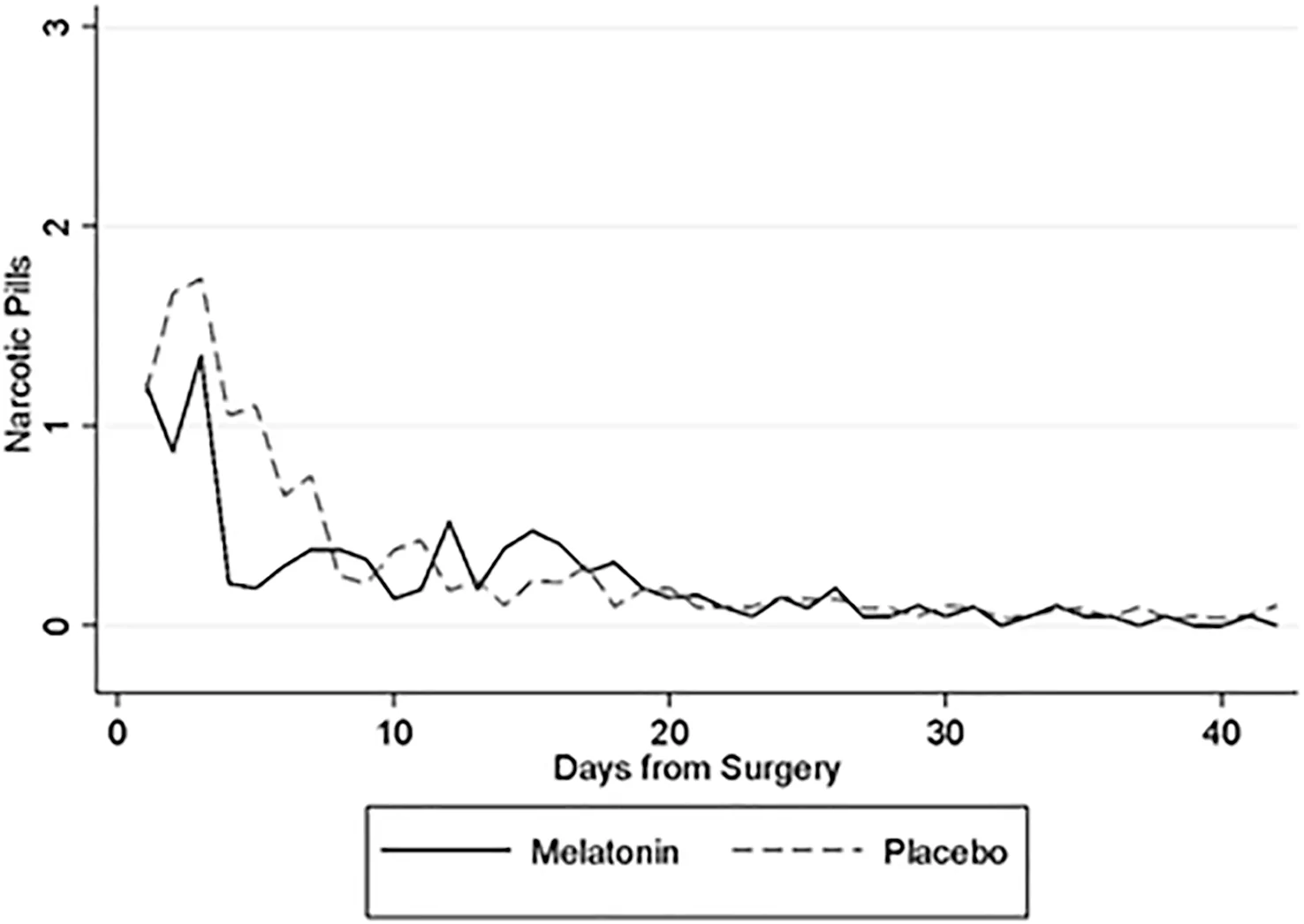
Daily narcotic pill use.

The use of melatonin did not improve sleep for the majority of metrics, nor did it lead to a decrease in narcotic use. Figure 2 shows daily non-tramadol narcotic use in the postoperative period for the melatonin and placebo group, with no significant difference (*P* = 0.36). When comparing daily questions about hours of sleep and sleep quality, there were no significant differences when all patients were included. When stratified by joint, the THA patients on melatonin had significantly more sleep, 0.75 h more per night over the 6 weeks, compared to placebo (*P* = 0.009) (Table 2). When comparing sleep and pain metrics (PSQI, SSS, sleep quality, VAS pain) over time for the melatonin vs placebo group, no significant differences were found except in preoperative PSQI (Table 3).

**Table 2:** Regression results.

	All		THA		TKA	
	Β (95% CI)	*p*-value	Β (95% CI)	*p*-value	Β (95% CI)	*p*-value
Hours of sleep	0.21 (−0.23, 0.65)	0.359	0.75 (0.18, 1.31)	0.009	−0.23 (−0.84, 0.39)	0.466
Quality of sleep	0.17 (−0.47, 0.80)	0.607	0.68 (−0.22, 1.59)	0.140	−0.27 (−1.08, 0.55)	0.524
Narcotics (number of pills per day)	−0.11 (−0.34, 0.12)	0.360	−0.18 (−0.56, 0.20)	0.353	−0.11 (−0.39, 0.18)	0.460

^*^Adjusted for age, body mass index, and pain at baseline.

THA: total hip arthroplasty; TKA: total knee arthroplasty.

**Table 3:** Sleep surveys over time by melatonin/placebo use.

	PSQI			SSS		
	Melatonin	Placebo		Melatonin	Placebo	
	Mean (std)	Mean (std)	*p*-value	Mean (std)	Mean (std)	*p*-value
Pre-op	5.4 (2.6)	8.0 (3.3)	0.0083	30.4 (6.9)	34.3 (15.0)	0.3873
2 weeks	6.2 (3.1)	7.3 (2.7)	0.2867	30.7 (7.8)	31.5 (11.8)	0.85
6 weeks	7.11 (4.6)	2.1 (3.2)	0.4381	34.1 (13.1)	29.9 (9.3)	0.3722
3 months	5.4 (3.8)	5.7 (2.6)	0.8551	27.4 (8.2)	28.1 (12.7)	0.8956
	Sleep quality			Pain		
	Melatonin	Placebo		Melatonin	Placebo	
	Mean (std)	Mean (std)	*p*-value	Mean (std)	Mean (std)	*p*-value
Pre-op	7.0 (1.8)	6.1 (2.3)	0.147	4.4 (2.2)	4.6 (2.4)	0.8238
2 weeks	6.6 (1.9)	6.0 (1.7)	0.3374	3.4 (2.5)	2.9 (2.2)	0.5431
6 weeks	7.2 (1.6)	7.4 (1.0)	0.6996	2.5 (2.2)	2.9 (2.7)	0.5772
3 months	7.2 (2.1)	7.6 (1.5)	0.5968	1.8 (2.1)	1.5 (1.6)	0.719

PSQI: Pittsburgh Sleep Quality Index; SSS: the Stanford Sleepiness Scale.

The secondary goal of this study was to better characterize the natural history of sleep disturbance after TJA. Table 4 shows the changes in sleep metrics and VAS Pain over time for all enrolled patients. Preoperatively, the average patient had a PSQI of 6.7, indicative of “poor” sleep. The PSQI was the same at 2 weeks, higher (i.e., worse sleep) at 6 weeks, and finally improved by 3 months postoperatively. The SSS, where a lower score is indicative of better sleep, followed a similar trend, with improvement not seen until 3 months. The sleep quality scale, with a higher score meaning improved sleep, showed worse sleep at 2 weeks, with gradual improvements at 6 weeks and 3 months. The VAS pain scores improved at every postoperative time point.

**Table 4:** Sleep metrics and VAS pain pre- and postoperative for all patients.

	PSQI	SSS	Sleep Quality	VAS Pain
	Mean (std)	Mean (std)	Mean (std)	Mean (std)
Pre-op	6.7 (3.2)	32.6 (12.1)	6.6 (2.1)	4.6 (2.4)
2 weeks	6.7 (2.9)	31.1 (9.8)	6.3 (1.8)	3.1 (2.2)
6 weeks	7.6 (3.9)	31.8 (11.1)	7.3 (1.3)	2.8 (2.4)
3 months	5.5 (3.2)	27.8 (10.0)	7.4 (1.8)	1.7 (1.8)

PSQI: Pittsburgh Sleep Quality Index; SSS: the Stanford Sleepiness Scale.

**Figure 3:**
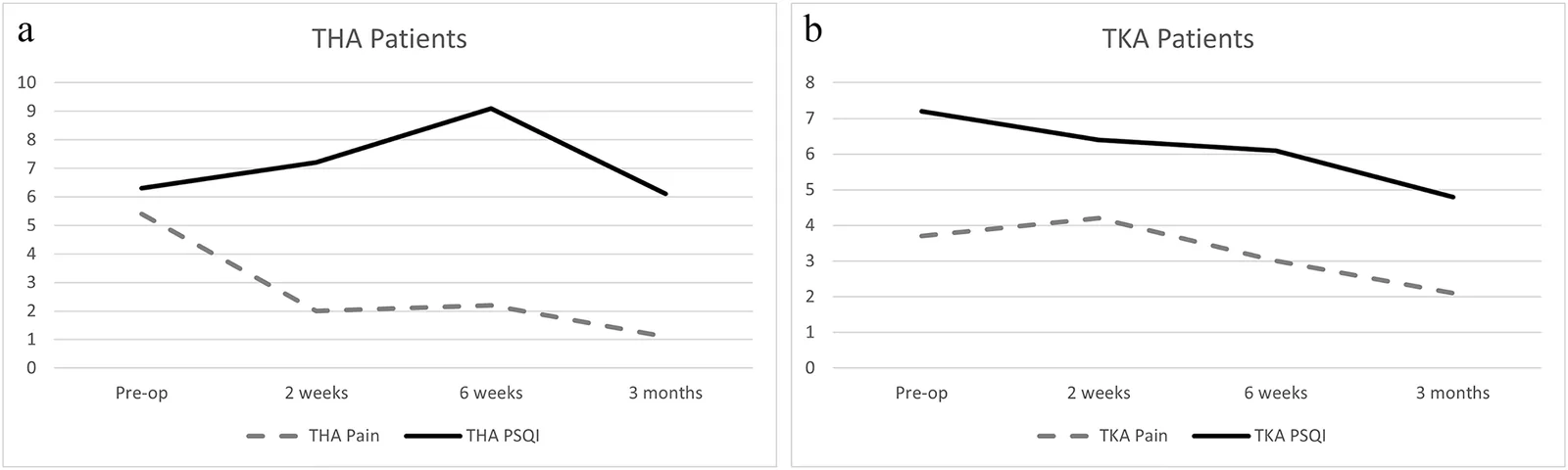
Sleep surveys by surgery type (a) total hip arthroplasty patients (b) total knee arthroplasty patients. PSQI: Pittsburgh Sleep Quality Index; TKA: total knee arthroplasty; THA: total knee arthroplasty.

We next compared THA to TKA patients, regardless of study arm. We found that for THA patients, VAS pain scores improved at every postoperative visit, but PSQI scores worsened at 2 weeks and 6 weeks before finally improving at 3 months. For TKA patients, the pain scores at 2 weeks were worse than preoperative scores, but sleep was already improved; PSQI scores improved at every postoperative time point, and by 3 months, TKA patients were no longer “poor” sleepers by PSQI criteria (Table 5 and Figure 3).

**Table 5:** PSQI and pain, THA vs TKA.

	THA		TKA	
	PSQI	Pain	PSQI	Pain
	Mean (std)	Mean (std)	Mean (std)	Mean (std)
Pre-op	6.3 (2.7)	5.4 (2.5)	7.2 (3.6)	3.7 (1.7)
2 weeks	7.2 (3.2)	2.0 (1.8)	6.4 (2.8)	4.2 (2.1)
6 weeks	9.1 (4.0)	2.2 (2.5)	6.1 (3.3)	3.0 (2.1)
3 months	6.1 (3.7)	1.1 (1.8)	4.8 (2.6)	2.1 (1.9)

PSQI: Pittsburgh Sleep Quality Index.

## Discussion

Prior literature has shown that joint replacement surgery can eventually lead to sleep improvement for osteoarthritis patients [11, 20, 21]. Unfortunately, patients must first navigate the immediate postoperative period, where sleep initially worsens. The first night after surgery, most patients have severe sleep deprivation and a loss of REM sleep [12]. Melatonin levels have been shown to decrease immediately after surgery with both spinal and general anesthetics [22]. Sleep structure is believed to improve over the first postoperative week, though some studies show disturbances in sleep quality for months following orthopedic surgery [11–13]. Elderly patients with low baseline sleep efficacy are at an especially increased risk for developing post op sleep disturbances.

There are two notable studies investigating the use of melatonin on perioperative orthopedic patients, which were published during our enrollment process. The first, by Tanner et al, was a prospective RCT of trauma patients [23]. They enrolled 84 patients at 2 weeks post op to either melatonin or a placebo for a 4-week duration. They found poor sleep scores at 2 and 6 weeks and did not find that melatonin had any effect on sleep scores or opioid use. Clarkson et al. had a similar study design to our own, with a prospective RCT of elective total joint patients [24]. They enrolled 118 patients who took melatonin or a placebo starting on postoperative day 1 for a total of 6 weeks after surgery. They found that melatonin had no effect on PSQI. Neither of these studies looked at preoperative sleep quality and quantity, nor did they look at longer-term sleep disturbances beyond the 6 weeks after surgery.

The findings in our study are consistent with what has been demonstrated previously in the limited orthopedic literature. Our primary outcome, the PSQI, is the most used validated sleep questionnaire. It determines good versus poor sleepers using information about sleep patterns over the preceding month. Scores greater than or equal to 5 label someone a “poor” sleeper, and scores over 10 are considered “severe” sleep disturbance. In the available literature prior to our study and the Clarkson et al. study, the average PSQI for osteoarthritis patients was around 9. The average pre-op PSQI scores of our patients (6.7) were nearly identical to those from the Clarkson et al. group (6.8), and they too saw sleep issues at 2 and 6 weeks after surgery [24]. However, they did not collect data for up to 3 months. They were better powered in their study and had similar results, showing that melatonin did not improve sleep scores. To our knowledge, prior to Tanner et al., no orthopedic study had looked at the effect of melatonin on postoperative opioid use [23]. Similar to their study, we found no decrease in daily opioid use with the addition of melatonin. Our data did show a significant increase in the hours of sleep in the THA melatonin group versus placebo, but no improvement in sleep scores overall or any other significant benefits from melatonin. Given the small sample size in our study and the lack of benefit from melatonin in similar, larger studies, we feel that this is likely attributable to sampling error.

One finding from our study that has not been previously described in the orthopedic literature is the difference between THA and TKA patients regarding postoperative sleep and pain. While it is well known that recovery, as measured by improved pain scores, is more rapid for total hip replacement than for total knee replacement, it was surprising to see more rapid restoration of sleep in this study’s TKA cohort [25, 26]. For THA patients, pain scores improved at every visit, but sleep did not improve until 3 months. TKA patients had worse pain at 2 weeks compared to preoperatively, but were already seeing an improvement in sleep scores. These patients continued to improve at every time point and by 3 months were, on average, no longer “poor” sleepers by PSQI criteria. This information could be useful in counseling patients about postoperative expectations regarding pain and sleep quality.

There are several limitations to this randomized controlled study. We were only able to include 48 patients in our analysis, rather than the 128 patients it was powered for. This was far fewer patients than we anticipated and our study was powered based upon larger numbers. This was obviously disappointing given the time and effort required by RCT. Two main factors played into this. First, the COVID pandemic overlapped with our study’s timing, and our institution’s requirement to restrict research coordinators in the clinic during this time interfered with enrollment. While we were still able to eventually screen over 1600 patients, this was over a much longer time than expected. Second, despite screening a large number of patients, most were not eligible due to preexisting underlying sleep issues and the current use of sleep aids. The rate of preexisting sleep issues excluded a surprisingly high rate of patients despite the knowledge that OA patients have poor sleep. Additionally, as is common with surveys, we relied on self-reported outcomes regarding sleep quantity and quality. Furthermore, we did not have a means to track patient compliance with medication use. For example, patients may have suspected they were receiving placebo pills and stopped taking them despite getting melatonin, thus missing out on the potential benefit of prolonged melatonin use. Another limitation is that we excluded patients with known sleep disorders, current sleep aid or calcium channel blocker use, and history of alcohol or drug abuse, and therefore our findings may not be generalizable to all patients. Finally, sleep is complex and difficult to study. Many different factors influence sleep, and most of these are out of our control as investigators, even in an RCT.

## Conclusion

This study validates and describes the natural history of sleep disturbances around TJA, both the preoperative and the prolonged postoperative sleep struggles that anecdotally we have noticed in our practices. Our study further proves that many TJA patients have poor sleep prior to surgery, which is frequently related to their osteoarthritis. Furthermore, sleep quality and quantity are negatively impacted after TJA surgery, often for several months. While TKA patients’ sleep improved by 2 weeks, THA patients still had worse sleep at 6 weeks and did not improve beyond baseline till 3 months after surgery. Interestingly, the sleep recovery did not correlate with pain scores or other PROs. The addition of perioperative melatonin led to more hours of sleep for THA patients but did not otherwise positively impact sleep quality, opioid use, or recovery in primary TJA patients. Further studies are required to research other sleep aides and modalities.

## Data Availability

De-identified data is available upon request for collaboration. Please contact the corresponding author.
